# Structural barriers to knowledge transfer and exchange among men and women in low-, middle- and high-income countries: an international cross-sectional study with vaccine researchers in 44 countries

**DOI:** 10.1186/s12961-021-00712-2

**Published:** 2021-04-12

**Authors:** Soha El-Halabi, Ronan McCabe, Birger C. Forsberg, Devy L. Elling, Ziad El-Khatib

**Affiliations:** 1 Department of Learning Informatics Management and Ethics, Karolinska Institutet, Stockholm, Sweden; 2 Department of Global Public Health, Karolinska Institutet, Stockholm, Sweden; 3 Department of Public Health Sciences, Stockholm University, Stockholm, Sweden; 4 Medical University of Vienna, Vienna, Austria; 5 World Health Programme, Université du Québec en Abitibi-Témiscamingue (UQAT), Québec, Canada

**Keywords:** Knowledge transfer and exchange, Evidence-informed practice, Research utilization, Gender, Barriers

## Abstract

**Background:**

Globally, women constitute 30% of researchers. Despite an increasing proportion of women in research, they are still less likely to have international collaborations. Literature on barriers to knowledge transfer and exchange (KTE) between men and women remains limited. This study aimed to assess perceived gender barriers to KTE activities in vaccination-related research in low-, middle- and high-income countries.

**Methods:**

This was a cross-sectional data assessment from a self-administered questionnaire distributed to researchers in the field of vaccination research. The administered questionnaire was developed and validated by WHO and McMaster University. Descriptive statistics were carried out. Structural factors of KTE were assessed using 12 statements measured with a five-point Likert scale, ranging from 1 (strongly disagree) to 5 (strongly agree). An index ranging from 12 to 60 points was created to assess structural factors of KTE, with higher score indicating fewer perceived barriers. Multivariable linear regression modelling was applied to examine the association between KTE barriers and gender.

**Results:**

A total of 158 researchers were included in the analysis. Regardless of gender and country of affiliation, researchers experienced challenges with respect to KTE activities; particularly factors related to the availability of human and financial resources and level of technical expertise among their target audience. We were also able to identify perceived facilitators among men and women, such as the presence of structures that link researchers and target audiences, the investment of target audiences in KTE efforts and the presence of stable contacts among target audiences. Our linear regression analysis showed that women perceived more barriers than men (*R*^2^ = 0.014; *B* = −1.069; 95% CI −4.035; 1.897).

**Conclusions:**

Men and women shared common perspectives on barriers to KTE. KTE activities could be strengthened by improving structural efforts to reduce gender differences and increase collaborations between researchers and their target audience.

**Supplementary Information:**

The online version contains supplementary material available at 10.1186/s12961-021-00712-2.

## Background

As one of the most cost-effective interventions in global health [1], vaccination has prevented approximately 3 million deaths annually [2]. Despite the numerous efforts being made to improve global vaccination coverage, WHO’s global vaccine targets were not met for the year 2020 [3]. Improved vaccination coverage could prevent 1.5 million deaths, representing 29% of under-five mortality. Despite this, the global burden of vaccine-preventable diseases (VPD) remains high [4]. In particular, inadequate vaccination remains a challenge in low- and middle-income countries (LMICs) [5]. While low vaccination coverage stems from a number of interrelated factors such as decreasing expenditure on vaccines, hesitancy and political instability [5–9], evidence-informed decision-making remains of crucial importance to addressing these challenges [10].

This is of particular importance in LMICs, as evidence generated at a local level is often ignored due to power imbalances or inadequate data quality [11]. Therefore, translation of extensive knowledge into relevant policies remains underutilized [12]. Evidence-informed decision-making can be promoted through knowledge transfer and exchange (KTE). KTE is an iterative process that links the three pillars of research, policy and practice to convert knowledge into policy [13]. KTE represents an exchange of knowledge between research producers and research users [14].

According to Lavis et al., KTE-related activities can be classified into four models, namely push efforts [15, 16], pull efforts [17, 18], exchange efforts and integrated efforts [15]. Push efforts are the identification of relevant policy research questions and the efforts of knowledge dissemination which are often researcher-led [15, 16], while pull efforts are defined as seeking information in order to support decision-makers in developing informed choices [17, 18]. With respect to exchange and integrated efforts, these are defined as collaboration between various actors and the cooperation of different stakeholders in conducting KTE-related activities [17].

The documentation of KTE activities in the field of vaccination research remains limited [19–21]. This could be due to having integrated KTE activities on the level of national policy and international initiatives that occur in a private fashion [22], or alternatively that KTE activities have not been carried out frequently due to inadequate structures to support KTE [11]. Structural barriers to KTE activities are barriers that occur on a system level. These include limited access to databases and research findings, financial limitations, limited administrative and infrastructural capacity, and the emergence of other priorities within the health system, as identified in previous studies [23–27]. Little has been documented on structural barriers to KTE in vaccination-related research. While there are some studies on structural barriers that health researchers face in KTE, specific literature on men and women’s perception of structural barriers to KTE remains limited [28].

The literature highlights the challenges that women face in the health sector and academia. Women are underrepresented in management, leadership and governance across the health and social care workforce. The High-Level Commission on Health Employment and Economic Growth considers gender biases to be limiting to the productivity, distribution, motivation and retention of female health workers, thus creating inefficiencies in the health system [29].

According to the United Nations Educational, Scientific and Cultural Organization (UNESCO) Institute for Statistics (2015), women constitute 30% of the world’s researchers [30]. Despite an increasing proportion of women researchers globally, they are still less likely to collaborate internationally [29]. Men publish more research papers on average than women [29]. Men are also more represented when it comes to first authorship. For every article with a woman as first author, there are approximately two articles with a male first author [31].

This study aimed to assess perceived gender barriers to KTE activities in vaccination-related research in low-, middle- and high-income countries. Specifically, this study aimed to (1) compare perceived structural barriers to and facilitators of KTE activities among men and women in vaccine-related research; and (2) investigate the association between gender and structural barriers to KTE activities in low-, middle- and high-income countries.

## Methods

### Study design

This study was based on cross-sectional data from an online self-administered questionnaire distributed to researchers between 28 March and 22 April 2018 (Additional file 1: Appendix 1). The questionnaire was developed and validated by WHO and McMaster University, Canada [32].

### Recruitment of participants

Participants were recruited based on the identification of vaccination-related articles obtained from PubMed using the search terms “(vaccinate* [MeSH Terms]) OR (immunize* [MeSH Terms])”. We screened the most recent publications for the period 1 January through 31 December 2017. The screening inclusion criteria were based on the availability of abstract and unique email addresses, the inclusion of human subjects, and articles written in English.

Based on these criteria, articles were included if the study population included children (< 18 years) or those in the proximity (e.g., parents, paediatrics, policies/programmes targeting children) or adults (> 18 years); conducted quantitative or qualitative analysis; and systematic reviews.

Additionally, articles were excluded if they were based on opinions or comments; were case reports; did not discuss VPD; did not include human subjects; were not written in English; or did not provide the email of the corresponding author.

Authors were invited to participate in the study via the email addresses obtained from the articles identified as relevant to the topic. In order to increase the response rate, reminders were sent on several occasions during a 1-month period (once per week during the first 2 weeks; twice a week during the third week; daily during the fourth week).

**Fig. 1:**
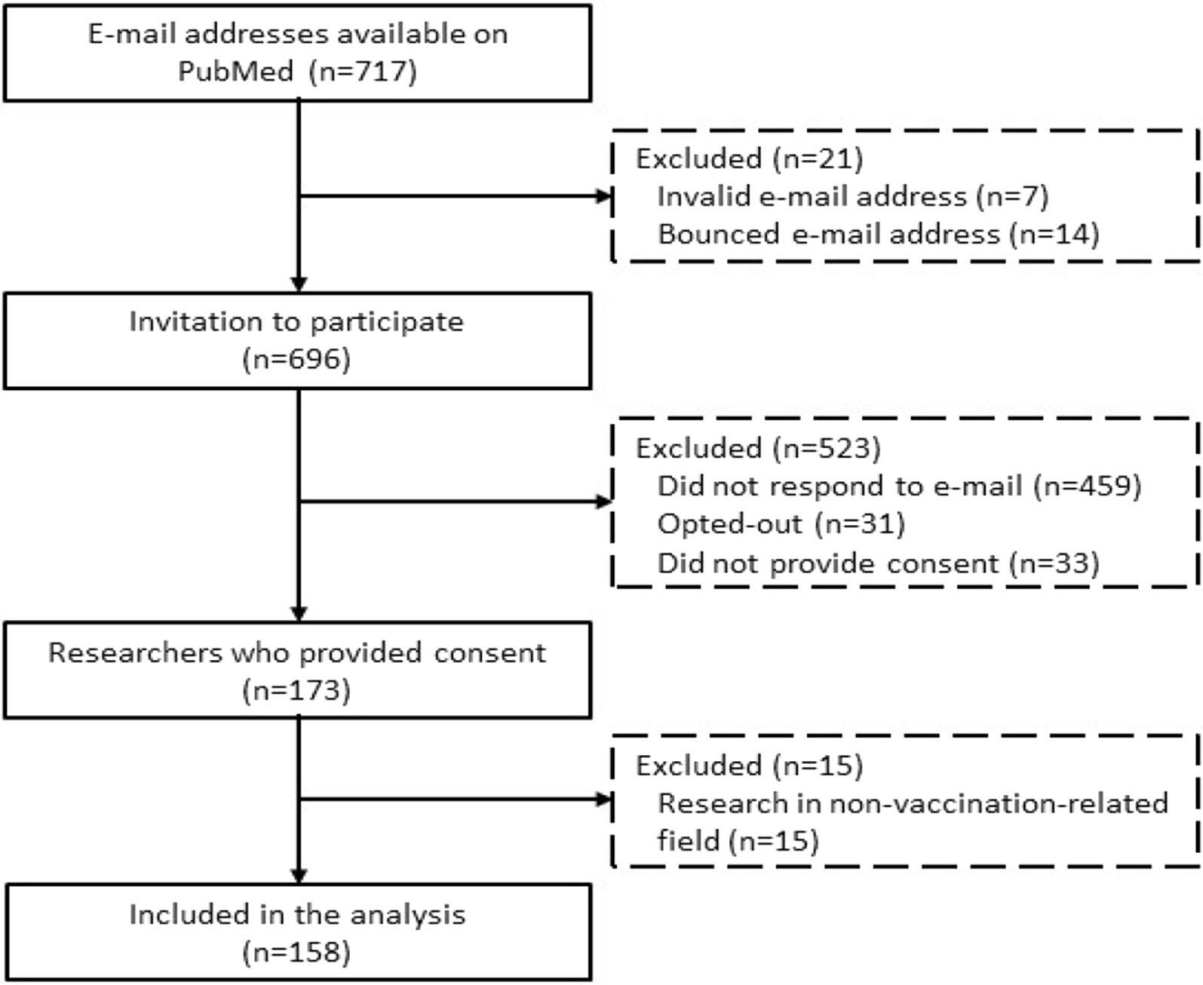
Schematic overview of recruitment of participants

During the recruitment process, a total of 717 researchers were identified and invited to participate in the survey. Of these, we included authors who had valid email addresses, provided consent and conducted research in a vaccination-related field. This resulted in a total number of 158 participants (Fig. 1).

### Variables

Structural factors of KTE were assessed using 12 statements measured with a five-point Likert scale, ranging from 1 (strongly disagree) to 5 (strongly agree). The statements were as follows:

1. The cost for translating research on the health topic into action was very low.
2. KTE activities could be paid for through research grants for which I was eligible to apply.
3. Structures and processes existed to link researchers and your target audiences.
4. Personal and organizational contacts among your target audiences were quite stable over time (e.g., low turnover among representatives and/or members of your target audiences).
5. Perceived crises in the health system drew attention away from research on the health topic.
6. Target audiences lacked the expertise for translating research on the health topic into action.
7. Target audiences had access to technical support for translating research on the health topic into action.
8. Target audiences created opportunities to develop joint research initiatives with them.
9. Target audiences did not make decisions about the health topic on the basis of research.
10. Target audiences invested financial and/or human resources in joint research initiatives.
11. Target audiences created events for knowledge transfer and exchange related to the health topic (e.g., forums that bring researchers and target audiences together for discussion).
12. Target audiences invested financial and/or human resources in knowledge transfer and exchange activities (e.g., hired staff to identify and make available relevant research).

The survey also included the variables of gender (men, women), year of birth, country of primary affiliation, education (medical doctor, bachelor’s degree, master’s degree, doctoral degree) and area of specialization (biomedical research, population and public health, clinical research, other). Based on the participants’ country of primary affiliation, the countries were divided into two income levels based on the country’s gross national income (GNI) per capita in 2018, according to the World Bank definition. Countries were categorized as LMICs if their GNI per capita was below or equal to US$ 12,235, while countries were categorized as high-income countries (HICs) if their GNI per capita was above US$ 12,235 [33]. In addition, age was calculated as the difference between 2018 and the year of birth.

### Statistical analysis

We tested the 12 statements for internal reliability using Cronbach’s alpha. The results showed some inconsistencies between the statements (Cronbach’s alpha = 0.504). In order to have a consistent measure of the items, the following items were reverse-coded: “Target audience lacked the expertise for translating research on the health topic into action”; “Target audience did not make decisions about the health topic on the basis of research”, increasing the internal consistency of the 12 statements (Cronbach’s alpha = 0.71) [34]. Further, we created an index with the 12 items, ranging from 12 to 60 points for KTE barriers, in which a lower score indicated more frequent experiences related to structural barriers regarding KTE activities.

To describe the study population, we computed descriptive statistics using Fisher’s exact test for the variables “country of primary affiliation”, “research specialization” and “educational attainment”. For the variable “structural factors of KTE”, the Mann–Whitney test was carried out to compare differences among men and women.

Multivariable linear regression analysis was applied to test the association between perceived KTE barriers and gender. In our model, the outcome (dependent variable) was a continuous variable on KTE score, and our independent variable was gender. We also included age and country of primary affiliation as covariates. The variables “gender” and “country of primary affiliation” were treated as binary variables. Dummy variables were created and coded as follows: male = 0, female = 1, HICs = 0 and LMICs = 1. Age was included as a continuous variable. Results are presented using beta coefficients and 95% confidence intervals (95% CI). We considered alpha *p* < 0.05 statistically significant. All statistical analyses were computed using SPSS statistical software version 25 (IBM Corp. Released 2017. IBM SPSS Statistics for Windows, Version 25.0. Armonk, NY: IBM Corp.). Our final analyses included participants who responded to questions related to barriers in implementing KTE activities (*n* = 54) only. Additionally, we conducted an analysis to examine differences between respondents and nonrespondents with regard to their sociodemographic characteristics (i.e., age, country of affiliation, educational attainment and area of research specialization). The only difference in relation to these variables was a larger proportion of women with doctoral degrees among the nonresponders group (Additional file 1: Appendix 2)."

## Results

### Description of participants

In our study, the majority of the study participants were men (*n* = 84; 53.0%), aged 37–47 years (men: *n* = 28; 33.3%; women: *n* = 30; 40.5%), had their primary affiliation in HICs (men: *n* = 61; 72.6%; women: *n* = 63; 85.1%), had a master’s degree (men: *n* = 26; 31.0%; women: *n* = 14; 18.9%), and had their research specialization in population and public health (men: 36.9%; women: *n* = 29 39.1%). The two gender populations were comparable; no statistically significant differences were observed (Table 1).

**Table 1:** Descriptive statistics by gender

	Men (*N* = 84)	Women (*N* = 74)	*p* value	Total (*N* = 158)
	*n* (%)	*n* (%)		
Age in years (*N* = 155)				
26–36	10 (11.9%)	13 (17.6%)		23
37–47	28 (33.3%)	30 (40.5%)		58
48–58	26 (31.0%)	20 (27.0%)		46
59–69	16 (19.0%)	10 (13.5%)		26
70+	2 (2.4%)	0 (0%)	0.44	2
Country of primary affiliation (*N* = 158)				
HICs	61 (72.6%)	63 (85.1%)		124
LMICs	23 (27.4%)	11 (14.9%)	0.08	34
Educational attainment (*N* = 71)				
Bachelor’s degree*	13 (15.5%)	12 (16.2%)		25
Master’s degree*	26 (31.0%)	14 (18.9%)		40
Doctoral degree*	0 (0%)	6 (8.1%)	0.25	6
Area of research specialization (*N* = 81)				
Biomedical research	2 (2.4%)	1 (1.4%)		3
Clinical research	8 (9.5%)	6 (8.1%)		14
Population and public health (including health policy and systems research)	31 (36.9%)	29 (39.1%)		60
Other	3 (3.6%)	1 (1.4%)	0.71	4

*Includes respondents who are medical doctors

### Structural factors of KTE^1^

In our study, we found structural factors perceived as barriers to KTE among men and women. More than half of men (*n* = 14; 53.9%) and women (*n* = 15; 53.6%) did not perceive their target audience to invest human and financial resources in KTE activities or in joint research initiatives (*n* = 11; 42.3% and *n* = 17; 60.7%). Another perceived barrier among men (*n* = 13; 50%) and women (*n* = 16; 57.2%) was lack of expertise among their target audience to translate research into action. In addition, 34.6% of men (*n* = 9) and 42.8% of women (*n* = 12) thought that their target audience lacked access to technical support to translate research into action. High costs for translating research into action was also a common perceived barrier among men (*n* = 12; 46.2%) and women (*n* = 14; 50%). Less than a quarter of men (*n* = 11; 42.3%) and women (*n* = 10; 35.7%) perceived that research grants for KTE activities were available. Further, less than a quarter of men (*n* = 11; 42.3%) and women (*n* = 11; 39.2%) perceived crises in the health system as a barrier to KTE (Table 2).

**Table 2:** Perceived barriers to KTE activities according to men and women

Barriers to KTE (*N* = 54)	Men (*N* = 26)	Women (*N* = 28)	*p* value
	*n* (%)	*n* (%)	
Target audiences invested financial and/or human resources in knowledge transfer and exchange activities (e.g., hired staff to identify and make available relevant research)			
Strongly disagree	4 (15.4)	5 (17.9)	
Disagree	10 (38.5)	10 (35.7)	
Neither agree nor disagree	8 (30.8)	10 (35.7)	
Agree	4 (15.4)	3 (10.7)	
Strongly agree	0 (0)	0 (0)	0.83
Target audiences lacked the expertise for translating research on the health topic into action			
Strongly disagree	0 (0)	1 (3.6)	
Disagree	8 (30.8)	3 (10.7)	
Neither agree nor disagree	5 (19.2)	8 (28.6)	
Agree	9 (34.6)	15 (53.6)	
Strongly agree	4 (15.4)	1 (3.6)	0.73
The cost for translating research on the health topic into action was very low			
Strongly disagree	6 (23.1)	6 (21.4)	
Disagree	6 (23.1)	8 (28.6)	
Neither agree nor disagree	10 (38.5)	6 (21.4)	
Agree	4 (15.4)	6 (21.4)	
Strongly agree	0 (0)	2 (7.1)	0.67
Target audiences invested financial and/or human resources in joint research initiatives			
Strongly disagree	3 (11.5)	8 (28.6)	
Disagree	8 (30.8)	9 (32.1)	
Neither agree nor disagree	10 (38.5)	7 (25)	
Agree	4 (15.4)	3 (10.7)	
Strongly agree	1 (3.8)	1 (3.6)	0.14
Target audiences had access to technical support for translating research on the health topic into action			
Strongly disagree	2 (7.7)	3 (10.7)	
Disagree	7 (26.9)	9 (32.1)	
Neither agree nor disagree	8 (30.8)	9 (32.1)	
Agree	7 (26.9)	4 (14.3)	
Strongly agree	2 (7.7)	3 (10.7)	0.50
KTE activities could be paid for through research grants for which I was eligible to apply			
Strongly disagree	1 (3.8)	5 (17.9)	
Disagree	8 (30.8)	9 (32.1)	
Neither agree nor disagree	6 (23.1)	4 (14.3)	
Agree	10 (38.5)	9 (32.1)	
Strongly agree	1 (3.8)	1 (3.6)	0.25
Perceived crises in the health system drew attention away from research on the health topic			
Strongly disagree	0 (0)	4 (14.3)	
Disagree	8 (30.8)	8 (28.6)	
Neither agree nor disagree	7 (26.9)	5 (17.9)	
Agree	7 (26.9)	9 (32.1)	
Strongly agree	4 (15.4)	2 (7.1)	0.28
Target audiences created opportunities to develop joint research initiatives with them			
Strongly disagree	1 (3.8)	6 (21.4)	
Disagree	6 (23.1)	6 (21.4)	
Neither agree nor disagree	11 (42.3)	7 (25)	
Agree	8 (30.8)	9 (32.1)	
Strongly agree	0 (0)	0 (0)	0.35
Target audiences did not make decisions about the health topic on the basis of research			
Strongly disagree	0 (0)	0 (0)	
Disagree	9 (34.6)	4 (14.3)	
Neither agree nor disagree	10 (38.5)	12 (42.9)	
Agree	7 (26.9)	9 (32.1)	
Strongly agree	0 (0)	3 (10.7)	0.06

We were also able to highlight some structural factors that men and women perceived as facilitators of KTE. About half of the men (*n* = 13; 50%) and women (*n* = 18; 64.2%) perceived the presence of stable contacts among their target audience as a facilitator to KTE. The presence of structures linking researchers to target audiences was also perceived as a facilitator among men (*n* = 12; 46.1%) and women (*n* = 12; 42.9%). The engagement of target audiences in KTE activities was perceived by some men (*n* = 12; 46.1%) and women (*n* = 11; 39.3%) as a facilitator (Table 3).

**Table 3:** Perceived facilitators of KTE activities among men and women

	Men (*N* = 26)	Women (*N* = 28)	*p* value
Facilitators of KTE (*N* = 54)	*n* (%)	*n* (%)	
Target audiences created events for knowledge transfer and exchange related to the health topic (e.g., forums that bring researchers and target audiences together for discussion)			
Strongly disagree	2 (7.7)	5 (17.9)	
Disagree	4 (15.4)	5 (17.9)	
Neither agree nor disagree	8 (30.8)	7 (25.0)	
Agree	11 (42.3)	10 (35.7)	
Strongly agree	1 (3.8)	1 (3.6)	0.38
Structures and processes existed to link researchers and your target audiences			
Strongly disagree	2 (7.7)	1 (3.6)	
Disagree	7 (26.9)	8 (28.6)	
Neither agree nor disagree	5 (19.2)	7 (25)	
Agree	11 (42.3)	11 (39.3)	
Strongly agree	1 (3.8)	1 (3.6)	0.98
Personal and organizational contacts among your target audiences were quite stable over time (e.g., low turnover among representatives and/or members of your target audiences)			
Strongly disagree	1 (3.8)	1 (3.6)	
Disagree	3 (11.5)	4 (14.3)	
Neither agree nor disagree	9 (34.6)	5 (17.9)	
Agree	11 (42.3)	16 (57.1)	
Strongly agree	2 (7.7)	2 (7.1)	0.51

### Index on KTE structural factors

**Fig. 2:**
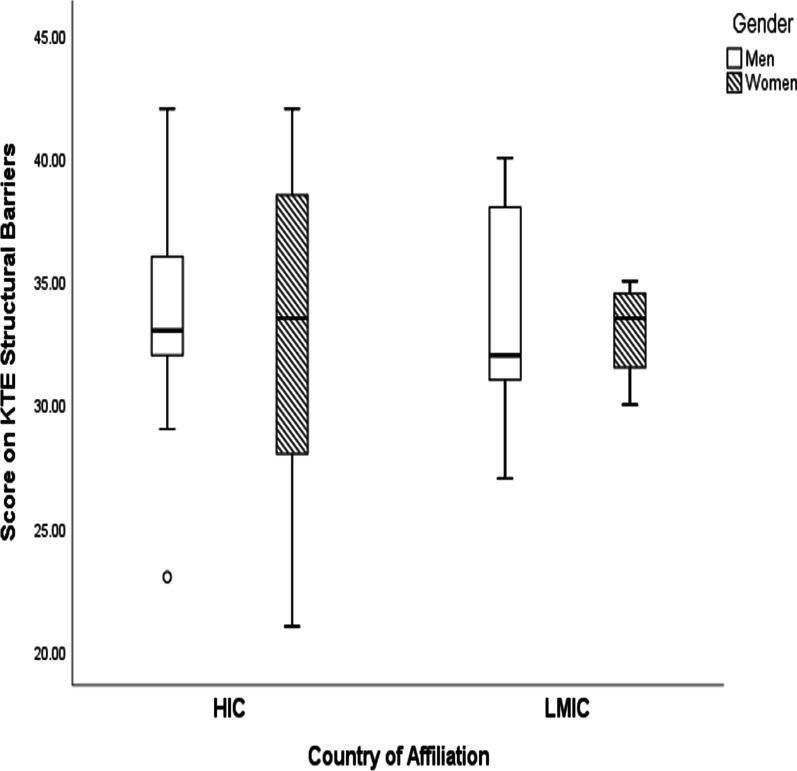
Score on KTE structural factors among men and women in HICs and LMICs

The mean score of the variable structural factors of KTE was highest among men affiliated with HICs (33.94; min–max: 23–42) and lowest among women affiliated with LMICs (33.0; min–max: 30–35). In our sample, women affiliated with HICs (32.88; min–max: 21–42) scored higher than women affiliated with LMICs but lower than men affiliated with LMICs (33.78; min–max: 27–40). There were no statistically significant differences among the mean scores calculated (*p* = 0.09) (Fig. 2).

### Association between perceived structural barriers for KTE activities and gender

Table 4 shows that, though not statistically significant, women perceived more structural barriers for KTE activities than men (*B*: −1.069; 95% CI: −4.035 to 1.897).

**Table 4:** Multivariable linear regression analysis for score on KTE structural factors

Unstandardized coefficients			*t*	*p* value	95% Confidence interval for *B*	
(Constant)	*B*	Std. error				
	32.735	3.332	9.823	0.000	26.038	39.431
Age	0.023	0.061	0.382	0.704	−0.099	0.146
Gender	−1.069	1.476	−0.724	0.472	−4.035	1.897
Country of affiliation	0.061	1.717	0.035	0.972	−3.391	3.512

## Discussion

This study aimed to compare and investigate the association between male and female vaccine researchers’ perceptions of structural barriers to and facilitators of KTE activities. Our results mostly reflect responses from vaccine researchers with master’s level degrees rather than those of doctorate level and above.

The mean scoring of men and women showed that researchers, regardless of gender or country of affiliation, experience some challenges when it comes to KTE activities. Men and women perceived factors related to the availability of human and financial resources and the level of technical expertise among target audiences as barriers. We were able to identify perceived facilitators among men and women, including the presence of structures linking researchers and target audiences, the investment of target audiences in KTE efforts and the presence of stable contacts among target audiences.

When we ran our linear regression model, we could see a relation, albeit nonsignificant, between gender and a reduction in score. While we were not able to identify specific studies tackling gender barriers and KTE, it may be explained through the literature exploring challenges that women face in academia [29, 30, 35]. A previous study indicated that male faculty members were able to positively engage in research and obtain funding, while women found it harder to balance between undergraduate education and research efforts [36]. Women not only publish less than men; they also still face challenges in attaining decision-making positions. Despite having more women in mid-management positions, men still dominate executive and full professorship positions globally [29]. This can be clearly seen in the field of health, where women still represent lower cadres of health workers despite constituting about 75% of the global health workforce [29]. However, this study did not show differences specifically in relation to KTE activities. This could be an interesting phenomenon to explore in future studies.

We found a substantial body of literature on barriers to KTE; however, they were conducted in different settings [37–41]. Additionally, we were unable to find any studies tackling barriers to KTE in relation to vaccination and gender. To our knowledge, our study presents a novel idea in the field of KTE and vaccination. It investigates KTE from the perspective of the research community, drawing comparisons between genders. It also contributes to the growing research on KTE in vaccination, which may still be considered as limited. Our study may also serve as a foundation for future research, as it encompasses a global scope.

This study had its own limitations as well. The external validity of the findings may be limited. Respondent withdrawal and missing data were observed in sections related to barriers. Despite having 158 respondents in total, 65.8% did not respond to questions related to structural factors. We suspect that the lengthy questionnaire was a contributing factor to this. Additionally, we did not ask the respondents about their current role, for example if they were actively working as medical professionals, at the university or at a healthcare facility/hospital or they were (post)-graduate students. This may have affected their response to the KTE-related activities, although we did define KTE in our survey. Another limitation in our study may be attributed to our selection process. The study excluded researchers who did not speak English and/or published in grey literature due to our questionnaire administration criteria. The questionnaire administered was originally developed for LMICs only. It also included only two options for the gender variable. While we did not have missing data for this variable, it may have been more convenient to not limit gender identities to man and woman only.

## Conclusion

This study did not highlight statistically significant differences between men and women in HICs and LMICs when it comes to vaccine-related KTE. Men and women shared common perspectives on barriers to KTE. The findings of this study show that more efforts on a structural level need to be carried out to strengthen KTE activities. Based on the results, it is important to invest in financial and human resources in KTE activities. These efforts should not be the sole responsibility of researchers. The target audience and decision-makers need to be more engaged in strengthening the implementation of KTE activities. Future research may examine the barriers to and facilitators of KTE at the organizational level.

## Supplementary Information

**Additional file 1**: Appendix 1. McMaster University/World Health Organization Questionnaire on Knowledge Transfer and Exchange in the Health Sector. Appendix 2. Nonrespondent analysis.

## Data Availability

The data sets used and/or analysed during the current study are available from the corresponding author on reasonable request.

## Abbreviations

KTE: Knowledge transfer and exchange
LMICs: Low- and -middle-income countries
VPD: Vaccine-preventable diseases
GNI: Gross national income
HICs: High-income countries
CI: Confidence interval

## Footnotes

1 In this section, we report aggregated responses of “agree” and “strongly disagree” as well as “disagree” and “strongly disagree”.
