# Supplementary material for: Structural barriers to knowledge transfer and exchange among men and women in low-, middle- and high-income countries: an international cross-sectional study with vaccine researchers in 44 countries
Source: Health Res Policy Syst. 2021 Apr 12;19:64. doi: 10.1186/s12961-021-00712-2 (PMC8042701; doi:10.1186/s12961-021-00712-2)
Supplement: Supplementary file 1 — Additional file 1: Appendix 1. McMaster University/World Health Organization Questionnaire on Knowledge Transfer and Exchange in the Health Sector. Appendix 2. Nonrespondent analysis. [file 12961_2021_712_MOESM1_ESM.docx]

**Appendix 1**

**McMaster University / World Health Organization Questionnaire on**

**Knowledge Transfer and Exchange in the Health Sector**

**Researcher Version – 11 May 2004**

| Many health researchers undertake a variety of activities with the aim that research will be considered and/or acted upon outside the scholarly community (i.e., by individuals other than researchers). Historically these efforts have had a variety of titles including: research transfer, technology transfer, communications, dissemination, guideline implementation, knowledge translation, and knowledge transfer and exchange. We use the term *knowledge transfer and exchange* (KTE) throughout this questionnaire for consistency.  The purpose of this questionnaire is to learn more about how you have undertaken and viewed these activities and about the context in which you undertook them.  Please check or circle the most appropriate response for each question. If you have specific comments on any issues raised in particular questions, please identify the question by number and add your comments in the space provided on the final page of the questionnaire. |
| --- |

i. Have you conducted any research on **[health topic]**?

□ Yes (Please continue)

□ No (If no, please return the questionnaire with only this box ticked)

| Hereafter we refer to research on **[health topic]** as “research on the health topic.” Please answer all subsequent questions with *only* this type of research in mind even if this research constitutes only a small proportion of the research with which you have been involved. Please do not consider research unrelated to the health topic. |
| --- |

ii. Have you undertaken one or more activities in the hope that research on the health topic will be considered and/or acted upon *outside* the scholarly community (i.e., by individuals other than researchers)?

□ Yes (Please continue)

□ No (If no, please complete Questions 20-23 only)

iii. Was your objective in undertaking these activities *exclusively* related to commercialization (e.g., patents)?

□ No (Please continue)

□ Yes (If yes, please complete Questions 20-23 only)

iv. Have you worked with or for one or more organizations and/or groups that, in addition to providing support for your research, undertook some knowledge transfer and exchange (KTE) activities related to the health topic in conjunction with you or on your behalf? If so, please list them here and circle the *one* primary organization with which you worked most closely on these KTE activities.

v. What were the time frames over which you a) conducted research on the health topic and b) undertook knowledge transfer and exchange (KTE) activities related to research on the health topic. Please circle all time frames that apply.

| **Before 1995**  **1** | | **1995-1997**  **2** | **1998-2000**  **3** | **2001-2003**  **4** | **2004**  **5** | | | | | |
| --- | --- | --- | --- | --- | --- | --- | --- | --- | --- | --- |
|  | Conducted research on the health topic | | | | | 1 | 2 | 3 | 4 | 5 |
|  | Undertook KTE activities related to the health topic | | | | | 1 | 2 | 3 | 4 | 5 |

| Please answer all questions about knowledge transfer and exchange (KTE) activities based on **both** your *usual* activities and your primary organization’s (i.e., the one you circled above) *usual* activities that were conducted in conjunction with you or on your behalf during the time frame in which you and/or your organization were undertaking these KTE activities, not what you and/or your organization considered doing or planned to do and not what your organization did not do in conjunction with you or on your behalf. |
| --- |

vi. Please indicate how often you and/or your organization undertook knowledge transfer and exchange activities related to the health topic for each of the following categories of potential users of research on the health topic.

| **Never**  **1** | | **Rarely**  **2** | **Occasionally**  **3** | **Frequently**  **4** | | **Always**  **5** | | | | |
| --- | --- | --- | --- | --- | --- | --- | --- | --- | --- | --- |
|  | General public and civil society groups | | | | 1 | | 2 | 3 | 4 | 5 |
|  | Patients and their families | | | | 1 | | 2 | 3 | 4 | 5 |
|  | Clinicians (e.g., nurses, doctors, etc.) | | | | 1 | | 2 | 3 | 4 | 5 |
|  | Managers in health-care institutions (e.g., hospitals), planning regions (e.g., health districts), non-governmental organizations, and third-party payers (e.g., health-care insurance companies) | | | | 1 | | 2 | 3 | 4 | 5 |
|  | Managers in donor agencies (e.g., European Community, Swedish International Development Agency) and international organizations (e.g., World Health Organization) | | | | 1 | | 2 | 3 | 4 | 5 |
|  | Managers in pharmaceutical and other biotechnology companies | | | | 1 | | 2 | 3 | 4 | 5 |
|  | Public policy-makers (i.e., elected officials, political staff, and civil servants) in local and national governments | | | | 1 | | 2 | 3 | 4 | 5 |
|  |  | | | |  | |  |  |  |  |

| Hereafter we refer to the potential users of research on the health topic for whom you and/or your organization *frequently or always* undertook knowledge transfer and exchange (KTE) activities as your “target audiences.” Please answer all questions about KTE activities with *only* these target audiences in mind. If you have two or more such target audiences and you conducted different KTE activities with different target audiences, please note these differences in the space provided on the final page of the questionnaire.  If you did not *frequently* or *always* undertake KTE activities for any of the above potential users of research on the health topic, please circle the *one* most important category of potential users to whom you and/or your organization *rarely* or *occasionally* undertook KTE activities, and please answer all questions about KTE activities with *only* this target audience in mind. |
| --- |

| In summary, please answer all questions keeping in mind:   1. research on the health topic only; 2. activities that were undertaken by you and/or by your organization working in conjunction with you or on your behalf; 3. usual activities during the time frame you and/or your organization were undertaking these activities; and 4. activities directed at your target audiences (defined as either the potential users of research for whom you and/or your organization frequently or always undertook KTE activities or the one most important category of potential users for whom you and/or your organization rarely or occasionally undertook KTE activities). |
| --- |

| Questions 1-10 ask how often you undertook particular knowledge transfer and exchange (KTE) activities, and the possible response categories range from *never* to *always*. When answering these questions, please keep in mind that how often you undertook each KTE activity may depend on how often it was feasible for you to do so, given the nature of the activity and the context in which you work.   - If you undertook a particular KTE activity whenever it was feasible to do so, please indicate:   - *always* if you undertook the activity every single time it was feasible or   - *frequently* if you did so almost every single time it was feasible. - If you undertook a particular KTE activity at least once but much less often than it was feasible to do so, please indicate:   - *occasionally* if you undertook the activity more often than not or   - *rarely* if you hardly ever did so. - If you never undertook a particular KTE activity whether it was feasible to do so or not, please indicate *never*. |
| --- |

**1)** Please indicate how often you (and/or your organization working in conjunction with you or on your behalf) performed each of these knowledge transfer and exchange (KTE) activities related to the health topic.

| **Never**  **1** | | **Rarely**  **2** | **Occasionally**  **3** | **Frequently**  **4** | **Always**  **5** | | | | | |
| --- | --- | --- | --- | --- | --- | --- | --- | --- | --- | --- |
|  | Provided reprints / copies of articles published in scientific journals to your target audiences (*not* including syntheses or formal systematic reviews of the research literature) | | | | | 1 | 2 | 3 | 4 | 5 |
|  | Provided full reports on research projects to your target audiences (*not* including syntheses or formal systematic reviews of the research literature) | | | | | 1 | 2 | 3 | 4 | 5 |
|  | Provided syntheses of the research literature to your target audiences  (*not* including formal systematic reviews of the research literature that follow explicit rules to reduce bias in searching the literature, identifying eligible articles, extracting data, etc.) | | | | | 1 | 2 | 3 | 4 | 5 |
|  | Provided formal systematic reviews of the research literature to your target audiences | | | | | 1 | 2 | 3 | 4 | 5 |
|  | Developed brief summaries of articles and/or research reports for your target audiences (*not* including brief summaries of syntheses and/or formal systematic reviews) | | | | | 1 | 2 | 3 | 4 | 5 |
| f. | Developed brief summaries of syntheses and/or formal systematic reviews of the research literature for your target audiences | | | | | 1 | 2 | 3 | 4 | 5 |
| g. | Developed messages for your target audiences that specified possible action (i.e., recommendations, take-home messages, actionable messages) | | | | | 1 | 2 | 3 | 4 | 5 |

**2)** Please indicate how often you (and/or your organization working in conjunction with you or on your behalf) performed each of these knowledge transfer and exchange (KTE) activities related to the health topic.

| **Never**  **1** | | **Rarely**  **2** | **Occasionally**  **3** | **Frequently**  **4** | **Always**  **5** | | | | | |
| --- | --- | --- | --- | --- | --- | --- | --- | --- | --- | --- |
|  | Obtained and/or updated contact information for your target audiences | | | | | 1 | 2 | 3 | 4 | 5 |
|  | Obtained and/or reviewed information about your target audiences (i.e., their needs and/or goals) | | | | | 1 | 2 | 3 | 4 | 5 |
|  | Developed reports, summaries or messages that used language appropriate to specific target audiences (e.g., non-technical language for the general public and civil society groups) | | | | | 1 | 2 | 3 | 4 | 5 |
|  | Developed reports, summaries or messages that were appealing to specific target audiences (e.g., graphics, colour, humour, and packaging) | | | | | 1 | 2 | 3 | 4 | 5 |
|  | Developed reports, summaries or messages that provided examples or demonstrations of how specific target audiences could use the research | | | | | 1 | 2 | 3 | 4 | 5 |
|  | Tailored the content of mailings or e-mails to specific target audiences | | | | | 1 | 2 | 3 | 4 | 5 |
|  | Tailored other aspects of your KTE approach to specific target audiences | | | | | 1 | 2 | 3 | 4 | 5 |

**3)** Please indicate how often you (and/or your organization working in conjunction with you or on your behalf) performed each of these knowledge transfer and exchange (KTE) activities related to the health topic.

| **Never**  **1** | | **Rarely**  **2** | **Occasionally**  **3** | **Frequently**  **4** | **Always**  **5** | | | | | |
| --- | --- | --- | --- | --- | --- | --- | --- | --- | --- | --- |
|  | Worked with KTE specialists in your organization | | | | | 1 | 2 | 3 | 4 | 5 |
|  | Reviewed the research literature about effective approaches to KTE | | | | | 1 | 2 | 3 | 4 | 5 |
|  | Reviewed information from websites, list-serves, etc. about effective approaches to KTE | | | | | 1 | 2 | 3 | 4 | 5 |
|  | Participated in KTE skill-building activities (e.g., conferences or courses about KTE) | | | | | 1 | 2 | 3 | 4 | 5 |
|  | Shared experiences with people performing KTE roles in other organizations like your own | | | | | 1 | 2 | 3 | 4 | 5 |
|  | Identified and worked with KTE specialists outside your organization | | | | | 1 | 2 | 3 | 4 | 5 |
|  | Identified and worked with knowledge brokers outside your organization (i.e., “people who bring researchers and their target audiences together and build relationships among them that make knowledge transfer and exchange more effective”) | | | | | 1 | 2 | 3 | 4 | 5 |
|  | Identified and worked with the most credible messengers for your target audiences (i.e., those who, regardless of their role or organization, are seen as credible by members of your target audience) | | | | | 1 | 2 | 3 | 4 | 5 |
|  | Developed relationships with print, radio and/or television journalists | | | | | 1 | 2 | 3 | 4 | 5 |

**4)** Please indicate how often you (and/or your organization working in conjunction with you or on your behalf) performed each of these knowledge transfer and exchange (KTE) activities related to the health topic.

| **Never**  **1** | | **Rarely**  **2** | **Occasionally**  **3** | **Frequently**  **4** | **Always**  **5** | | | | | |
| --- | --- | --- | --- | --- | --- | --- | --- | --- | --- | --- |
|  | Provided *at cost* and upon request articles, reports, syntheses and/or formal systematic reviews for your target audiences | | | | | 1 | 2 | 3 | 4 | 5 |
|  | Provided *free* upon request (but not through a website) articles, reports, syntheses and/or formal systematic reviews for your target audiences | | | | | 1 | 2 | 3 | 4 | 5 |
|  | Provided *through a website* articles, reports, syntheses and/or formal systematic reviews for your target audiences | | | | | 1 | 2 | 3 | 4 | 5 |
|  | Provided *free* upon request (but not through a website) *brief summaries* of articles, reports, syntheses formal systematic reviews and/or *messages* that specified possible action for your target audiences | | | | | 1 | 2 | 3 | 4 | 5 |
|  | Provided *through a website* brief summaries and/or messages for your target audiences | | | | | 1 | 2 | 3 | 4 | 5 |
|  | Provided *on a website* dedicated entry-points / sections for specific target audiences | | | | | 1 | 2 | 3 | 4 | 5 |
|  | Posted to a *list-serve* brief summaries and/or messages for your target audiences | | | | | 1 | 2 | 3 | 4 | 5 |
|  | Mailed or e-mailed to your target audiences *notices* that new material of potential interest to them had been posted to a website | | | | | 1 | 2 | 3 | 4 | 5 |
|  | Mailed or e-mailed to your target audiences articles, reports, syntheses and/or formal systematic reviews *without an explicit request* from some or all members of your target audiences | | | | | 1 | 2 | 3 | 4 | 5 |
|  | Mailed or e-mailed to your target audiences (but not through a list-serve or newsletter) *brief summaries* of articles, reports, syntheses and/or formal systematic reviews and/or *messages* that specified possible action for your target audiences *without an explicit request* from some or all members of your target audiences | | | | | 1 | 2 | 3 | 4 | 5 |
|  | Mailed or e-mailed to your target audiences a *newsletter* containing brief summaries and/or messages | | | | | 1 | 2 | 3 | 4 | 5 |
|  | Mailed or e-mailed to your target audiences a *newsletter* containing dedicated sections for specific target audiences | | | | | 1 | 2 | 3 | 4 | 5 |
|  | Submitted *media releases* to print, radio and/or television journalists | | | | | 1 | 2 | 3 | 4 | 5 |
|  | Accepted requests from journalists to participate in interviews or debates | | | | | 1 | 2 | 3 | 4 | 5 |
|  | Published research in non-scholarly publications read by your target audiences (e.g., general interest magazines for the general public, or publications produced by professional associations for clinicians or managers) | | | | | 1 | 2 | 3 | 4 | 5 |

**5)** Please indicate how often you (and/or your organization working in conjunction with you or on your behalf) interacted (e.g., through teleconferences or face to-face meetings involving a small number of people) with representatives and/or members of your target audiences *in each of the following stages of the research process* for all research projects related to the health topic with which you have been involved.

| **Never**  **1** | | **Rarely**  **2** | **Occasionally**  **3** | **Frequently**  **4** | **Always**  **5** | | | | | |
| --- | --- | --- | --- | --- | --- | --- | --- | --- | --- | --- |
|  | Interacted when developing a specific research question, objectives or hypothesis | | | | | 1 | 2 | 3 | 4 | 5 |
|  | Interacted when establishing the preferred research design and methods | | | | | 1 | 2 | 3 | 4 | 5 |
|  | Interacted when executing the research | | | | | 1 | 2 | 3 | 4 | 5 |
|  | Interacted when analyzing / interpreting the research findings | | | | | 1 | 2 | 3 | 4 | 5 |
|  | Interacted when developing research products (e.g., research reports, brief summaries and/or messages) | | | | | 1 | 2 | 3 | 4 | 5 |
|  | Interacted when undertaking KTE activities for your target audiences | | | | | 1 | 2 | 3 | 4 | 5 |
|  | Interacted when responding to individual queries resulting from your research products and/or KTE efforts | | | | | 1 | 2 | 3 | 4 | 5 |

**6)**Please indicate how often you (and/or your organization working in conjunction with you or on your behalf) interacted with representatives and/or members of your target audiences about research on the health topic in the following contexts *outside of the research process per se*.

| **Never**  **1** | | **Rarely**  **2** | **Occasionally**  **3** | **Frequently**  **4** | **Always**  **5** | | | | | |
| --- | --- | --- | --- | --- | --- | --- | --- | --- | --- | --- |
|  | Interacted through government-sponsored meetings involving your target audiences | | | | | 1 | 2 | 3 | 4 | 5 |
|  | Interacted through an expert committee or group involving your target audiences | | | | | 1 | 2 | 3 | 4 | 5 |
|  | Interacted through conferences and workshops involving your target audiences | | | | | 1 | 2 | 3 | 4 | 5 |
|  | Interacted through public hearings or testimonies involving your target audiences | | | | | 1 | 2 | 3 | 4 | 5 |
|  | Interacted through formal private or public networks involving your target audiences | | | | | 1 | 2 | 3 | 4 | 5 |
|  | Interacted through events organized by you and/or your organization | | | | | 1 | 2 | 3 | 4 | 5 |
|  | Interacted through events organized by your target audiences | | | | | 1 | 2 | 3 | 4 | 5 |
|  | Interacted through events organized by print, radio and/or television journalists | | | | | 1 | 2 | 3 | 4 | 5 |
|  | Interacted through informal conversations with your target audiences | | | | | 1 | 2 | 3 | 4 | 5 |
|  | Interacted through events organized by bilateral, regional or international organizations (e.g., World Health Organization) | | | | | 1 | 2 | 3 | 4 | 5 |
|  | Interacted through other mechanism - please specify: ______________________________ | | | | | 1 | 2 | 3 | 4 | 5 |

**7)** Please indicate how often you (and/or your organization working in conjunction with you or on your behalf) performed each of these types of assessments or evaluations of your knowledge transfer and exchange (KTE) activities related to the health topic.

| **Never**  **1** | | **Rarely**  **2** | **Occasionally**  **3** | **Frequently**  **4** | **Always**  **5** | | | | | |
| --- | --- | --- | --- | --- | --- | --- | --- | --- | --- | --- |
|  | Assessed the perceived *usefulness* of research products made available to your target audiences (e.g., reports, brief summaries, and messages) | | | | | 1 | 2 | 3 | 4 | 5 |
|  | Assessed the perceived *usefulness* of your and/or your organization’s KTE activities | | | | | 1 | 2 | 3 | 4 | 5 |
|  | Assessed any changes in your target audiences’ *awareness* of the research on the health topic that may be attributable to your KTE activities | | | | | 1 | 2 | 3 | 4 | 5 |
|  | Assessed any changes in your target audiences’ *knowledge* of research on the health topic that may be attributable to your KTE activities | | | | | 1 | 2 | 3 | 4 | 5 |
|  | Assessed any changes in your target audiences’ *attitudes* towards research on the health topic that may be attributable to your KTE activities | | | | | 1 | 2 | 3 | 4 | 5 |
|  | Assessed any changes in your target audiences’ *self-reported* *behaviour* that may be attributable to your KTE activities | | | | | 1 | 2 | 3 | 4 | 5 |
|  | Assessed any changes in your target audiences’ *actual* (i.e., objectively measured) *behaviour* that may be attributable to your KTE activities | | | | | 1 | 2 | 3 | 4 | 5 |

**8)** Please indicate how often you (and/or your organization working in conjunction with you or on your behalf) performed each of these knowledge transfer and exchange (KTE) activities to make it easier for your target audiences to obtain research on the health topic when they needed it.

| **Never**  **1** | | **Rarely**  **2** | **Occasionally**  **3** | **Frequently**  **4** | **Always**  **5** | | | | | |
| --- | --- | --- | --- | --- | --- | --- | --- | --- | --- | --- |
|  | Provided access to a searchable database of articles, reports, syntheses, and or formal systematic reviews on the health topic | | | | | 1 | 2 | 3 | 4 | 5 |
|  | Provided access to a searchable database of brief summaries of articles, reports, syntheses and/or formal systematic reviews and/or messages that specified possible action for your target audiences | | | | | 1 | 2 | 3 | 4 | 5 |
|  | Clearly identified in websites, newsletters, and other communication vehicles the specific individual(s) who was involved in the development of a report, summary and/or message and who could answer questions about it | | | | | 1 | 2 | 3 | 4 | 5 |
|  | Clearly identified in websites, newsletters, and other communication vehicles the specific individual(s) who could answer questions about research on the health topic whether or not they were involved in conducting it | | | | | 1 | 2 | 3 | 4 | 5 |
|  | Maintained some reserve capacity (i.e., financial and/or human resources that can be redirected when required) to conduct short-term research projects in response to requests from your target audiences | | | | | 1 | 2 | 3 | 4 | 5 |
|  | Other – please specify: _____________________________________________________ | | | | | 1 | 2 | 3 | 4 | 5 |

**9)** Please indicate how often you (and/or your organization working in conjunction with you or on your behalf) performed activities (e.g., conducted workshops or seminars) to increase the capacity of your target audiences to use research on the health topic.

| **Never**  **1** | | **Rarely**  **2** | **Occasionally**  **3** | **Frequently**  **4** | **Always**  **5** | | | | | |
| --- | --- | --- | --- | --- | --- | --- | --- | --- | --- | --- |
|  | Developed capacity of target audiences to *acquire* research on the health topic through searchable databases (e.g., MedLine, Cochrane, African Index Medicus, Index Medicus for Eastern Mediterranean Region, Index Medicus for South-East Asia Region, Latin American and Caribbean Center on Health Sciences Information (LILACS)) | | | | | 1 | 2 | 3 | 4 | 5 |
|  | Developed capacity of target audiences to *assess* the quality and applicability of research on the health topic | | | | | 1 | 2 | 3 | 4 | 5 |
|  | Developed capacity of target audiences to *adapt* research on the health topic to increase its perceived relevance (e.g., by linking it to local issues) | | | | | 1 | 2 | 3 | 4 | 5 |
|  | Developed capacity of target audiences to *apply* research on the health topic (e.g., by combining research with other types of information relevant to the decisions they face) | | | | | 1 | 2 | 3 | 4 | 5 |

**10)** Please indicate how often you (and/or your organization working in conjunction with you or on your behalf) performed each of these knowledge transfer and exchange (KTE) activities related to the health topic.

| **Never**  **1** | | **Rarely**  **2** | **Occasionally**  **3** | **Frequently**  **4** | **Always**  **5** | | | | | |
| --- | --- | --- | --- | --- | --- | --- | --- | --- | --- | --- |
|  | Established and/or maintained long term *partnerships* related to the health topic with representatives and/or members of your target audiences (e.g., through an advisory board) | | | | | 1 | 2 | 3 | 4 | 5 |
|  | Involved representatives and/or members of your target audiences in conducting a *needs assessment* for your target audiences | | | | | 1 | 2 | 3 | 4 | 5 |
|  | Involved representatives and/or members of your target audiences in establishing the *overall direction of research* on the health topic conducted by you and/or your research organization | | | | | 1 | 2 | 3 | 4 | 5 |
|  | Involved representatives and/or members of your target audiences in establishing the *overall direction of KTE activities* related to the health topic undertaken by you and/or your research organization | | | | | 1 | 2 | 3 | 4 | 5 |
|  | Involved representatives and/or members of your target audiences in *assessing the progress of research* on the health topic conducted by you and/or your research organization | | | | | 1 | 2 | 3 | 4 | 5 |
|  | Involved representatives and/or members of your target audiences in *assessing the progress of KTE activities* on the health topic undertaken by you and/or your research organization | | | | | 1 | 2 | 3 | 4 | 5 |

**11)** Please indicate how often during a *typical 12-month period* you (and/or your organization working in conjunction with you or on your behalf) performed each of these KTE activities related to the health topic.

| **Never**  **1** | | **About 3-4 times during the last 12 months**  **2** | **About every month**  **3** | **Weekly**  **4** | **More than once a week**  **5** | | | | | |
| --- | --- | --- | --- | --- | --- | --- | --- | --- | --- | --- |
|  | Provided reprints / copies of articles published in scientific journals to your target audiences (*not* including syntheses or formal systematic reviews) | | | | | 1 | 2 | 3 | 4 | 5 |
|  | Provided syntheses of the research literature to your target audiences  (*not* including formal systematic reviews of the research literature that follow explicit rules to reduce bias in searching the literature, identifying eligible articles, extracting data, etc.) | | | | | 1 | 2 | 3 | 4 | 5 |
|  | Provided formal systematic reviews of the research literature to your target audiences | | | | | 1 | 2 | 3 | 4 | 5 |
|  | Developed brief summaries of articles and/or research reports for your target audiences (*not* including brief summaries of syntheses and/or formal systematic reviews) | | | | | 1 | 2 | 3 | 4 | 5 |
|  | Developed messages for your target audiences that specified possible action (e.g., recommendations, take-home messages, actionable messages) | | | | | 1 | 2 | 3 | 4 | 5 |
|  | Developed reports, summaries or messages that provided examples or demonstrations of how specific target audiences could use the research | | | | | 1 | 2 | 3 | 4 | 5 |
|  | Mailed or e-mailed to your target audiences a *newsletter* containing brief summaries and/or messages | | | | | 1 | 2 | 3 | 4 | 5 |
|  | Accepted requests from journalists (radio, television, newpaper, etc) to participate in interviews or debates | | | | | 1 | 2 | 3 | 4 | 5 |

1. Please indicate how often during a *typical 12-month* period you (and/or your organization working in conjunction with you or on your behalf) interacted with representatives and/or members of your target audiences about research on the health topic in the following contexts *outside of the research process per se*.

| **Never**  **1** | | **About 3-4 times during the last 12 months**  **2** | **About every month**  **3** | **Weekly**  **4** | **More than once a week**  **5** | | | | | |
| --- | --- | --- | --- | --- | --- | --- | --- | --- | --- | --- |
|  | Interacted through government-sponsored meetings involving your target audiences | | | | | 1 | 2 | 3 | 4 | 5 |
|  | Interacted through events organized by you and/or your organization | | | | | 1 | 2 | 3 | 4 | 5 |
|  | Interacted through informal conversations with your target audiences | | | | | 1 | 2 | 3 | 4 | 5 |

**13)** Please estimate the percentage of your own total work time during a *typical 12-month period* in which you were involved in knowledge transfer and exchange (KTE) activities related to the health topic that you spent performing KTE activities related to the health topic. _______ %

**14)** Please indicate the degree to which you agree or disagree with the following statements concerning the state of research knowledge when you were involved in research on the health topic and in knowledge transfer and exchange (KTE) activities related to the health topic.

| **Strongly Disagree**  **1** | | **Disagree**  **2** | **Neither agree nor disagree**  **3** | **Agree**  **4** | **Strongly agree**  **5** | | | | | |
| --- | --- | --- | --- | --- | --- | --- | --- | --- | --- | --- |
|  | No synthesis was possible because there was too much research available | | | | | 1 | 2 | 3 | 4 | 5 |
|  | One or more syntheses were available for use by your target audiences | | | | | 1 | 2 | 3 | 4 | 5 |
|  | No synthesis was possible because research was confidential | | | | | 1 | 2 | 3 | 4 | 5 |
|  | One or more syntheses were available but not in the language(s) spoken by your target audiences | | | | | 1 | 2 | 3 | 4 | 5 |
|  | No synthesis was possible because research was out of date | | | | | 1 | 2 | 3 | 4 | 5 |
|  | One or more syntheses were available but not in language appropriate to specific target audiences (e.g., non-technical language for the general public and civil society groups) | | | | | 1 | 2 | 3 | 4 | 5 |
|  | No synthesis was possible because research was lacking on important issues | | | | | 1 | 2 | 3 | 4 | 5 |

**15)** Please indicate the degree to which you agree or disagree with the following statements concerning the barriers to and facilitators of knowledge transfer and exchange (KTE) when you were involved in research on the health topic and in knowledge transfer and exchange (KTE) activities related to the health topic.

| **Strongly Disagree**  **1** | | **Disagree**  **2** | **Neither agree nor disagree**  **3** | **Agree**  **4** | **Strongly agree**  **5** | | | | | |
| --- | --- | --- | --- | --- | --- | --- | --- | --- | --- | --- |
|  | The cost for translating research on the health topic into action was very low | | | | | 1 | 2 | 3 | 4 | 5 |
|  | KTE activities could be paid for through research grants for which I was eligible to apply | | | | | 1 | 2 | 3 | 4 | 5 |
|  | Structures and processes existed to link researchers and your target audiences | | | | | 1 | 2 | 3 | 4 | 5 |
|  | Personal and organizational contacts among your target audiences were quite stable over time (e.g., low turnover among representatives and/or members of your target audiences) | | | | | 1 | 2 | 3 | 4 | 5 |
|  | Perceived crises in the health system drew attention away from research on the health topic | | | | | 1 | 2 | 3 | 4 | 5 |
|  | Target audiences lacked the expertise for translating research on the health topic into action | | | | | 1 | 2 | 3 | 4 | 5 |
|  | Target audiences had access to technical support for translating research on the health topic into action | | | | | 1 | 2 | 3 | 4 | 5 |
|  | Target audiences did not make decisions about the health topic on the basis of research | | | | | 1 | 2 | 3 | 4 | 5 |
|  | Target audiences created opportunities to develop joint research initiatives with them | | | | | 1 | 2 | 3 | 4 | 5 |
|  | Target audiences invested financial and/or human resources in joint research initiatives | | | | | 1 | 2 | 3 | 4 | 5 |
|  | Target audiences created events for knowledge transfer and exchange related to the health topic (e.g., forums that bring researchers and target audiences together for discussion) | | | | | 1 | 2 | 3 | 4 | 5 |
|  | Target audiences invested financial and/or human resources in knowledge transfer and exchange activities (e.g., hired staff to identify and make available relevant research) | | | | | 1 | 2 | 3 | 4 | 5 |

**16)** Please indicate whether you had access to the following sources of information when you were involved in research on the health topic and in knowledge transfer and exchange (KTE) activities related to the health topic.

| Yes | | No | Don’t Know | | | |
| --- | --- | --- | --- | --- | --- | --- |
|  | Had access to at least five scientific journals indexed in the Health InterNetwork Access to Research Initiative (HINARI) (full text paper or full text electronic)) | | | Yes | No | Don’t know |
|  | Had access to at least five scientific journals indexed in *other* international reference databases (e.g., Medline, PubMed or the equivalent for your field) (full text paper or full text electronic) | | | Yes | No | Don’t know |
|  | Had access to at least five scientific journals published locally, nationally or regionally (full text paper or full text electronic) | | | Yes | No | Don’t know |
|  | Had access to the internet / web at least once a month to conduct and download searches | | | Yes | No | Don’t know |
|  | Had access to a personal computer with a functional internet connection at all times to conduct and download searches | | | Yes | No | Don’t know |

**17)** Please indicate the degree to which you agree or disagree with the following statements concerning support for knowledge transfer and exchange (KTE) activities within your organization when you were involved in research on the health topic and in KTE activities related to the health topic.

| **Strongly Disagree**  **1** | | **Disagree**  **2** | **Neither agree nor disagree**  **3** | **Agree**  **4** | **Strongly agree**  **5** | | | | | |
| --- | --- | --- | --- | --- | --- | --- | --- | --- | --- | --- |
|  | The translation of research on the health topic into action was hampered by a lack of academic rewards for KTE activities | | | | | 1 | 2 | 3 | 4 | 5 |
|  | The translation of research on the health topic into action was helped by requirements within my organization to publish findings | | | | | 1 | 2 | 3 | 4 | 5 |
|  | The translation of research on the health topic into action was helped by the mix of researchers and target audiences within my organization | | | | | 1 | 2 | 3 | 4 | 5 |
|  | My organization made available financial and human resources to assist me with KTE activities | | | | | 1 | 2 | 3 | 4 | 5 |
|  | My organization assumed responsibility for undertaking KTE activities on my behalf | | | | | 1 | 2 | 3 | 4 | 5 |
|  | My organization was not seen as a credible source of research on the health topic | | | | | 1 | 2 | 3 | 4 | 5 |

**18)** Please indicate the degree to which you agree or disagree with the following statements concerning changes over time in support for research on the health topic and for knowledge transfer and exchange (KTE) activities related to the health topic.

| **Strongly Disagree**  **1** | | **Disagree**  **2** | **Neither agree nor disagree**  **3** | **Agree**  **4** | **Strongly agree**  **5** | | | | | |
| --- | --- | --- | --- | --- | --- | --- | --- | --- | --- | --- |
|  | When I began conducting research on the health topic, the *health research environment in my country* was supportive of individuals who *conduct research* on the health topic | | | | | 1 | 2 | 3 | 4 | 5 |
|  | Over the time that I conducted research on the health topic, the *health research environment in my country* became more supportive of individuals who *conduct research* on the health topic | | | | | 1 | 2 | 3 | 4 | 5 |
|  | When I began conducting research on the health topic, the *health research environment in my country* was supportive of individuals who *undertook KTE activities* related to the health topic | | | | | 1 | 2 | 3 | 4 | 5 |
|  | Over the time that I undertook KTE activities related to the health topic, the *health research environment in my country* became more supportive of individuals who *undertook KTE activities* on the health topic | | | | | 1 | 2 | 3 | 4 | 5 |
|  | When I began conducting research on the health topic, *my organization* was supportive of individuals who *conduct research* on the health topic | | | | | 1 | 2 | 3 | 4 | 5 |
|  | Over the time that I conducted research on the health topic, *my organization* became more supportive of individuals who *conduct research* on the health topic | | | | | 1 | 2 | 3 | 4 | 5 |
|  | When I began conducting research on the health topic, *my organization* was supportive of individuals who *undertook KTE activities* related to the health topic | | | | | 1 | 2 | 3 | 4 | 5 |
|  | Over the time that I undertook KTE activities related to the health topic, *my organization* became more supportive of individuals who *undertook KTE activities* on the health topic | | | | | 1 | 2 | 3 | 4 | 5 |

**19)** Please indicate the degree to which you agree or disagree with the following statements concerning your research on the health topic at the time you were conducting it and your views about who should be responsible for knowledge transfer and exchange (KTE) activities.

| **Strongly Disagree**  **1** | | **Disagree**  **2** | **Neither agree nor disagree**  **3** | **Agree**  **4** | **Strongly agree**  **5** | | | | | |
| --- | --- | --- | --- | --- | --- | --- | --- | --- | --- | --- |
|  | My research was not considered relevant by target audiences | | | | | 1 | 2 | 3 | 4 | 5 |
|  | My research coincided with my country’s priorities (e.g., with a National Research Agenda) | | | | | 1 | 2 | 3 | 4 | 5 |
|  | My research coincided with the needs and expectations of target audiences | | | | | 1 | 2 | 3 | 4 | 5 |
|  | My research lacked credibility among target audiences | | | | | 1 | 2 | 3 | 4 | 5 |
|  | My research was not yet ready for use | | | | | 1 | 2 | 3 | 4 | 5 |
|  | Researchers who conduct research on the health topic are primarily responsible for KTE activities related to the health topic | | | | | 1 | 2 | 3 | 4 | 5 |
|  | Target audiences for research on the health topic are primarily responsible for KTE activities related to the health topic | | | | | 1 | 2 | 3 | 4 | 5 |
|  | Both researchers and target audiences are jointly responsible for KTE activities related to the research topic | | | | | 1 | 2 | 3 | 4 | 5 |

**20)** What was your area of research specialization when you were conducting research on the health topic? Please select only one.

□ Biomedical research (e.g., to understand biology and mechanisms, to develop new therapeutic agents)

□ Clinical research (e.g., to improve diagnosis, to trial new therapeutic regimens, to improve patients’ quality of life)

□ Health policy and systems research (e.g., to improve health service delivery, to identify and quantify the impact of user charges)

□ Population and public health (e.g., to identify and quantify risk factors, to develop effective prevention strategies)

□ Other - please specify:

**21)** What type of graduate or post-graduate degree(s) have you completed, when and in what country did you obtain the degree(s)?

| **Degree** | **Name of degree**  **(e.g., MSc, MD, PhD)** | **Year obtained** | **Country obtained** |
| --- | --- | --- | --- |
| Health professional degree  (e.g., MD, nursing) |  |  |  |
| First masters degree |  |  |  |
| Second masters degree |  |  |  |
| Doctorate degree |  |  |  |
| Other graduate degree  (Please specify) |  |  |  |

**22)** What is your year of birth?

**23)** What is your gender? □ Male □ Female

**24)** Please summarize in point form the most salient findings from your *own* research on the health topic and, if possible, please provide a reference for the findings.

Reference (if available):

**25)** Please summarize in point form the most salient findings from the body of *all available* research on the health topic as it existed at the time you were conducting research on the health topic and undertaking KTE activities related to the health topic and, if possible, please provide a reference for the findings.

Reference (if available):

**26)** Please list the five most important knowledge transfer and exchange (KTE) activities that you performed for each of clinicians and policymakers even if they were not your principal target audiences. If you performed no KTE activities for either or both group, please indicate this.

Clinicians Policymakers

1) _______________________________________ 1) _______________________________________

2) _______________________________________ 2) _______________________________________

3) _______________________________________ 3) _______________________________________

4) _______________________________________ 4) _______________________________________

5) _______________________________________ 5)

**27)** Would you be willing to let us contact you again if other questions emerge from this research?

□ Yes □ No

**Thank you for completing the questionnaire.**

Please return the completed questionnaire in the large envelope provided.

As a small token of our appreciation, we are pleased to provide you and your organization with access to full-text articles from over 2000 journals from the world's leading biomedical publishers through the Health InterNetwork Access to Research Initiative (HINARI). If your organization does not already have access to HINARI, simply complete the electronic form, or ask your librarian to complete the electronic form, which is available at
<http://www.healthinternetwork.org/src/registration.php?lang=en>.

As well, we are pleased to provide your organization’s library with a comprehensive set of current WHO publications including the World Health Report. To receive these publications, simply complete the attached request form, place it in the small envelope addressed to Emmanuel Guindon of the World Health Organization, and seal the small envelope. Please insert this sealed envelope along with your completed questionnaire in the large return envelope addressed to the local investigator leading this study and seal the large envelope. Please be assured that your request form will be kept separate from your completed questionnaire.

ID #:

(Your responses will be kept confidential and data will not be reported in ways that could potentially identify you or your organization.)

**Additional Thoughts (Optional)**

Do you have any comments regarding any of the questions?

Do you want to note any differences in how you and/or your organization performed knowledge transfer and exchange (KTE) activities for different target audiences?

(If the space provided is insufficient to accommodate all your ideas, please feel free to attach additional pages.)

**Appendix 2. Non-respondent analysis**

|  | **Men**  **(N=58)** | | **Women**  **(N=46)** | | **p-value** | **Total**  **(N=104)** |
| --- | --- | --- | --- | --- | --- | --- |
|  | n | (%) | n | (%) |  |  |
| **Country of affiliation (N=104)** |  |  |  |  |  |  |
| HIC | 44 | (75.9%) | 39 | (84.8%) |  | 83 |
| LMICs | 14 | (24.1%) | 7 | (15.2%) | 0.32 | 21 |
| **Educational Attainment (N=27)** |  |  |  |  |  |  |
| Bachelor* | 4 | (6.8%) | 3 | (6.6%) |  | 7 |
| Master’s degree* | 8 | (14%) | 1 | (2.2%) |  | 9 |
| Doctorate Degree* | 5 | (8.5%) | 6 | (13.1%) | 0.5 | 11 |
| **Research specialization (N= 29)** |  |  |  |  |  |  |
| Biomedical research | 1 | (1.7%) | 1 | (2.2%) |  | 2 |
| Clinical research | 3 | (5.2%) | 3 | (6.5%) |  | 6 |
| Population and Public Health** | 12 | (20.7%) | 6 | (13%) |  | 18 |
| Other | 3 | (5.2%) | 0 | --- | 0.55 | 3 |
| **Mean age (SD)** **(N=103)** | 48.4 | (10.55) | 43.46 | (9.11) | 0.33 | --- |

**: Includes respondents who are medical doctors | **: Includes health policy and systems research*
